# FISHIS: Fluorescence *In Situ* Hybridization in Suspension and Chromosome Flow Sorting Made Easy

**DOI:** 10.1371/journal.pone.0057994

**Published:** 2013-02-28

**Authors:** Debora Giorgi, Anna Farina, Valentina Grosso, Andrea Gennaro, Carla Ceoloni, Sergio Lucretti

**Affiliations:** 1 ENEA – Italian National Agency for New Technologies, Energy and Sustainable Economic Development, CASACCIA Research Center, Rome, Italy; 2 DAFNE – Department of Agriculture, Forestry, Nature and Energy, University of Tuscia, Viterbo, Italy; Universidad Miguel Hernández de Elche, Spain

## Abstract

The large size and complex polyploid nature of many genomes has often hampered genomics development, as is the case for several plants of high agronomic value. Isolating single chromosomes or chromosome arms via flow sorting offers a clue to resolve such complexity by focusing sequencing to a discrete and self-consistent part of the whole genome. The occurrence of sufficient differences in the size and or base-pair composition of the individual chromosomes, which is uncommon in plants, is critical for the success of flow sorting. We overcome this limitation by developing a robust method for labeling isolated chromosomes, named Fluorescent *In situ* Hybridization In suspension (FISHIS). FISHIS employs fluorescently labeled synthetic repetitive DNA probes, which are hybridized, in a wash-less procedure, to chromosomes in suspension following DNA alkaline denaturation. All typical A, B and D genomes of wheat, as well as individual chromosomes from pasta (*T. durum* L.) and bread (*T. aestivum* L.) wheat, were flow-sorted, after FISHIS, at high purity. For the first time in eukaryotes, each individual chromosome of a diploid organism, *Dasypyrum villosum* (L.) Candargy, was flow-sorted regardless of its size or base-pair related content. FISHIS-based chromosome sorting is a powerful and innovative flow cytogenetic tool which can develop new genomic resources from each plant species, where microsatellite DNA probes are available and high quality chromosome suspensions could be produced. The joining of FISHIS labeling and flow sorting with the Next Generation Sequencing methodology will enforce genomics for more species, and by this mightier chromosome approach it will be possible to increase our knowledge about structure, evolution and function of plant genome to be used for crop improvement. It is also anticipated that this technique could contribute to analyze and sort animal chromosomes with peculiar cytogenetic abnormalities, such as copy number variations or cytogenetic aberrations.

## Introduction

Eukaryotic genomes are partitioned into chromosomes, and chromosome numbers vary between species from two to many hundreds [1]. Genomes can be investigated by a number of methodologies, some of which analyze total genomic DNA in bulk, whereas others preserve chromosomal individuality. Next Generation Sequencing (NGS) is a current example of the first approach [2], while cytogenetic and flow cytometry (FCM) are examples of the latter [3]. Plant genomics would necessitate the joining of both methodologies to overcome the huge task of assembling hybrid genomes and to represent the vast diversity existing into the species, and that cannot be shown up by the sequencing of a single individual genome. The understanding of the intra-species genetic variability would require *de novo* sequencing of several single genomes instead of the re-sequencing of the same genotype [4]. Even with the powerful third-generation sequencing technologies, this effort would be costly and difficult to apply for large plant polyploid genomes. The improved chromosome approach we propose can be used to reduce the whole-genome complexity, opening an easy access to single chromosomes from several and different species.

### Flow cytometry as an analytical and preparative technique

Flow cytometry, accompanied by sorting, permits isolation of individual chromosomes for further study, and generates highly pure and chromosome-specific DNA preparations. FCM involves the passage of chromosome suspensions through the focus of intense light sources, typically lasers, using one or two DNA-specific fluorochromes to provide fluorescent signals related to the DNA content and base-pair composition of the individual chromosomes. The end products are “flow karyotypes” providing one- or two-dimensional representations of the distributions of the different chromosomes (histograms or dot plot, respectively). In the first case, individual chromosomes form peaks within the distribution, the individual peaks representing differences in chromosome size, whereas, in the second, the individual chromosomes fall into clusters, separated both by DNA content and by differences in A–T/G–C contents as detected by base pair-specific fluorochromes. Chromosome peaks or clusters can then be automatically sorted to generate highly pure single chromosome preparations. Human genomics took early advantage of this technology, since the differences both in the DNA content and base pair composition detected by the A–T and C–G specific fluorochromes were sufficient to allow direct sorting of 21 of the 23 autosomes; this provided the chromosome-specific DNA necessary to initiate the project of sequencing the human genome [5]. Ideally, in a flow karyotype, each peak or cluster within the DNA distributions should represent a single chromosome. However, in plants, this is a rare situation. For many plant species, particularly crops of major economic significance, there are no such differences in chromosome sizes and DNA contents and base pair composition within the genomes. Thus, usually only one or few chromosomes can be identified and separated by flow sorting, while others cluster together forming composite peaks [6], [7].

### Plant chromosome flow sorting

As of now, methods for chromosome isolation and flow sorting have been reported for 22 plant species including major cereal crops and wild relatives [7], [8], [9]. The separation of a genome into its chromosomal components offers an effective means to generate a full genome sequence of large-genome species, often polyploids such as bread wheat, since it greatly simplifies the process of sequence assembly [8]. The International Research Initiative for Wheat Genome Sequencing Consortium IWGSC (http://www.wheatgenome.org) is taking advantage of flow sorting individual chromosomes and their arms [10], [11] to address the enormous complexity of the 16 Gbp bread wheat polyploid genome, which comprises three homeologous genomes containing over 80% repetitive DNA sequences [12]. So far, the standard wheat DNA flow karyotype has allowed purification of only one chromosome, namely 3B, the sequencing of which was accomplished by a clone-by-clone shotgun approach [11].

The remaining twenty chromosomes can be flow-sorted thanks to the availability of special cytogenetic stocks, containing specific chromosomes or chromosome arms that differ in size from the standard complement. These were developed from the laboratory line [13] of bread wheat cv ‘Chinese Spring’ (CS) and are currently used by the Consortium to sort individual chromosome arms [14] and to construct large insert of bacterial artificial chromosome (BAC) libraries [15]. It is unlikely that this demanding feat will ever be repeated in any other crop, or in specific cultivars of wheat that carry agronomically important genes of top value for breeding. Therefore, even in wheat as in most of the eukaryotes, the wider gene pool is currently closed to the approach of flow sorting individual chromosomes.

### FISH labeling of chromosomes in suspension

A novel strategy for specifically labeling individual chromosomes in suspension would be highly desirable as it would improve the resolution of flow sorting and expand the range of the chromosome approach to genomics to wide gene pools and many species [8]. Since flow sorting uses fluorescence signals to discriminate chromosomes, such a labeling strategy would have to involve *in situ* fluorescent hybridization using either chromosome-specific DNA sequences, or repetitive DNAs with chromosome-specific distribution patterns. Importantly, it requires that the level of the hybridization signal be detectable, which currently eliminates from consideration all low fluorescence intensity single-copy sequences. Nevertheless, satellites, microsatellites (or Simple Sequence Repeats – SSR) and transposable elements are widely distributed throughout genomes [16], [17] and have an excellent track record of use in cytogenetic characterization and identification of individual chromosomes [18], [19], [20], [21], [22].

Many efforts have been made to apply the classic FISH labeling procedure to nuclei and chromosomes in suspension both in human [23] and plant samples [24], [25] with generally unsatisfactory results. FISH involves denaturation of the probe and target DNAs and annealing under stringent enough conditions to provide specific and reproducible hybridization. Chromosomes in suspension tend to disintegrate or clump together when subjected to the heat denaturation conditions required for FISH. Attempts to combine FISH on isolated plant chromosomes in suspension followed by FCM have not been successful and no reports have been published of an effective flow cytometry analysis and sorting of FISH labeled plant chromosomes.

### A new FISH labeling technique for chromosomes in suspension

Here we describe a new method, termed “Fluorescence *In situ* Hybridization In Suspension” or FISHIS, which makes possible the coupling of the high discriminatory capabilities of FISH labeling and the high-throughput of flow cytometry analysis and flow sorting to isolate pure chromosome and nuclei fractions. FISHIS is a wash-less method [26] that relies on readily available fluorescently labeled DNA repetitive sequences (e.g. SSR) and employs alkaline DNA denaturation [27], [28]. By the use of flow sorting, we show that the method can discriminate, and hence purify: i) the entire chromosome set of a single genome from the homeologous one in polyploid pasta and bread wheat; ii) a number of the chromosomes of bread and pasta wheat, and iii) the entire chromosome complement of the diploid wild wheat relative *Dasypyrum villosum* (L.).

The FISHIS method extends the analytical and preparative power of flow cytometry to virtually all the individual chromosomes, generating new opportunities for the genomics approach to complex and valuable genomes.

## Materials and Methods

### Cell cycle synchronization and preparation of suspensions of chromosomes and nuclei

Grains were soaked in aerated water for 8–24 h and germinated on moist filter paper for two days in the dark at 19±1°C (root length 2–3 cm). Synchronization of the root tip cell cycle was by exposure to 1.25 mM hydroxyurea for 18 h, followed by immersion in aerated Hoagland's solution [29] for 4 h for pasta wheat and *D. Villosum* and for 4.5 h for bread wheat. Cell division was blocked at metaphase by a 2 h treatment with 2.5 µM amiprophos-methyl, and the resulting metaphase-arrested chromosomes were elongated and dispersed within the cytoplasm by an overnight incubation in ice water. To prepare chromosome suspensions [29], roots were excised and fixed in 3% (v/v) formaldehyde in 1x Tris-HCl pH 7.5 at 5±1°C for 20 mins and rinsed three times in 1x Tris-HCl pH 7.5 at 5±1°C for 5 mins. The distal 1 mm of a set of 50 roots was homogenized in 1 ml LB01 lysis buffer (Ultraturrax T10 with G5 generator, IKA, Germany) into polystyrene tubes (Falcon 2054) and the resulting homogenate filtered through a 36 μm nylon mesh to remove debris [30]. Nuclear suspensions were obtained by the same procedure omitting cell cycle synchronization.

### DNA Probes

Eurofins MWG Operon (Ebersberg, Germany) synthesized all the DNA probes: 5′ -FITC-(GAA)_7_-3′-FITC, 5′ -Cy3-(AG)_12_, 5′ -Cy3-(AAT)_7_ and 5′-Cy3-(AAC)_5._ The HPLC desalted oligonucleotides were dissolved at 1 µg/µl in 10 mM Tris, 1 mM EDTA.

### Non-denaturing FISH (ND-FISH)

Fast labeling by FISH in non denaturing conditions was performed on metaphase spreads according to Cuadrado *et al.* [31], with minor modifications: 50 ng of the (GAA)_7_ probe suspended in 30 µl of 2XSSC (300 mM sodium chloride, 0.3 mM trisodium citrate) were flushed on the slide and incubated at room temperature for 1 h; after incubation, followed by washing for 10 mins in 4XSSC with 0.2% Tween20, samples were counterstained with DAPI (4′,6-diamidino-2-phenylindole) and mounted in an antifade solution.

### FISHIS – Alkaline Denaturation of DNA

**Figure 1:**
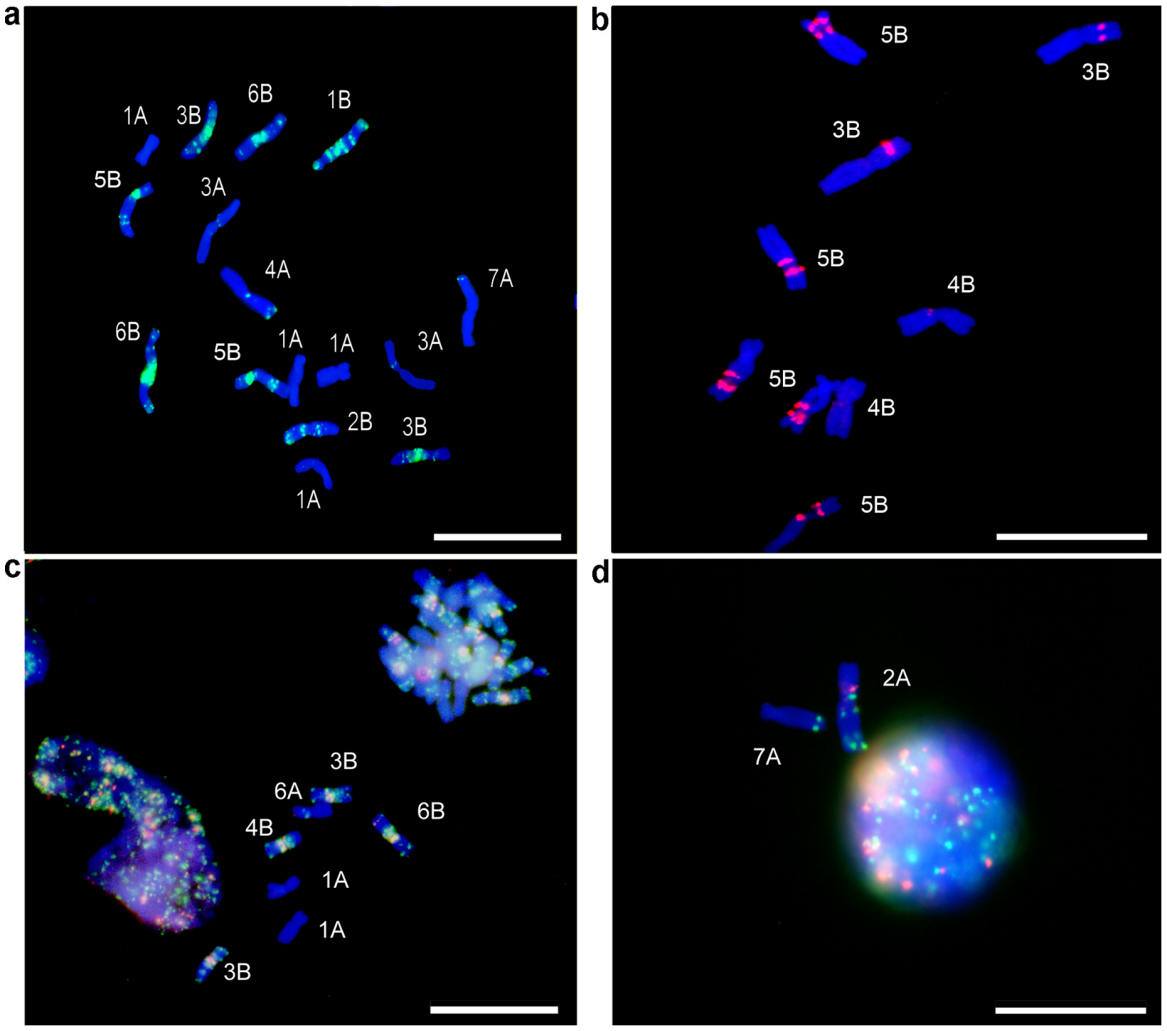
FISHIS of pasta wheat cv Creso chromosome suspensions. a) Chromosome suspensions hybridized with (GAA)_7_-FITC; b) flow-sorted chromosomes 3B, 4B and 5B following hybridization with (AG)_12_-Cy3; c) (GAA)_7_-FITC and (AAT)_7_-Cy3 dual labelled chromosomes and nuclei; d) pasta wheat chromosomes and a nucleus after (GAA)_7_-FITC and (AAC)_5_-Cy3 dual labeling. Bar  = 10 µm.

The optimal pH for DNA denaturation was determined experimentally by the addition of NaOH to a 150 µl aliquot of suspended chromosomes (2×10^6^ chromosomes/ml LB01). The pH measurements were performed with an ISFET micro pH probe (Hatch PHW17-SS) inserted into the sample tube. The extent of DNA denaturation over the pH range 8.0–13.8 was assessed as a time course (0–60 mins) by labeling with 36 µM Acridine Orange (AO) followed by flow cytometry [32] (AO stains single-stranded DNA red and double-stranded DNA green). Flow data were collected as single DNA histograms and dot plots plotting the ratio of ssDNA/dsDNA fluorescence against the dsDNA fluorescence for 104 chromosomes per sample, removing debris and aggregates from the forward scatter signal versus a DAPI fluorescence dot plot. The HPCV (Half Peak Coefficient of Variation: the standard deviation (s) of the fluorescent intensity of a population of chromosomes expressed as a percentage of the mean (m) intensity (CV  =  s/m•100) measured at 50% peak height) was taken as an index for chromosome morphology and DNA integrity [33]. The optimum denaturation treatment was set at pH 13 for 20 mins, followed by the addition of 1M Tris-HCl pH 7.4 and maintaining the suspension on ice for 1 min to return to pH****8.0. After denaturation, yield and morphology of chromosomes were evaluated by FCM (**Figure S1**) and microscope observation (**Figure 1**).

### FISHIS – Fluorescent labeling

The oligonucleotides were dissolved in 2XSSC at the concentration of 1 ng/µl and added to the sample at the lowest concentration at which fluorescent emission of the sample did not increase in intensity if the concentration was doubled. FISHIS-labeled chromosomes were analyzed by flow cytometry, and the concentration of 160 ng/ml was selected for labeling (**Figure S1c**). The labeling reaction was for 1 h at room temperature, without any washing or centrifugation. After hybridization, samples were diluted 1∶1 with LB01, counter-stained with 7 µM DAPI and analyzed by flow cytometry (300 µl final volume). For chromosome identification by fluorescence microscopy, 4 µl chromosome suspension was mounted in 30% LB01 and 70% Vectashield (v/v) (Vector Labs, Burlingame, CA) containing 7 µM DAPI.

### Flow Cytometry and Chromosome Sorting

**Figure 2:**
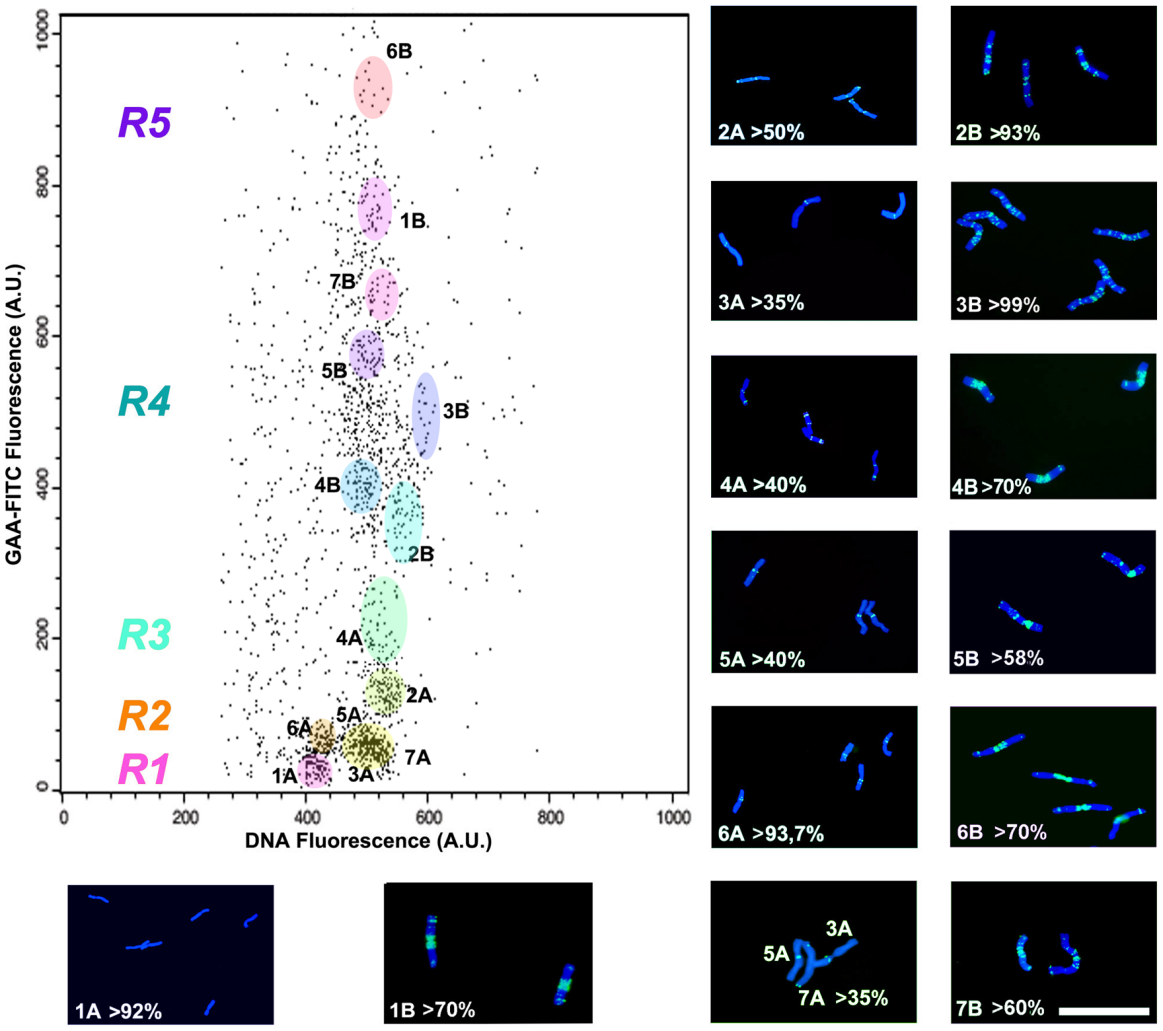
Biparametric dot plot analysis of pasta wheat cv Creso chromosomes. The fluorescence intensity emissions from chromosomes stained with DAPI (DNA content) and labeled by FISHIS with GAA-FITC are joint together into a bi-parametric dot plot where each dot represents a single particle (blue: DNA stained by DAPI; green: (GAA)_7_-FITC labeling). Similar particles with a similar fluorescence emission are clustered and can then be enclosed into a sorting region for flow sorting and single-type chromosome isolation (colored regions). Panels showing the chromosome content from each relevant dot plot sorting region display various purity levels. The sorting purity is presented as a percentage of the main sorted chromosome in respect to the total number of the sorted population. Chromosome region distribution is directly proportional to the whole intensity of the fluorescence hybridization pattern. Different colored regions R1–R5 were used to assess the MESF (Molecules of Equivalent Soluble Fluorescein) values (**Figure S5**). The (GAA)_7_ oligonucleotides hybridize less with the A-genome chromosomes than the B-genome ones. As expected, the A-genome chromosomes are found within regions R1–R3 and the B-genome chromosomes are enclosed in regions R4 and R5. Bar  = 10 µm.

All chromosome analysis and sorting were performed on a dual laser FACS Vantage SE flow cytometer (BD Bioscience, San Jose, CA). Flow cytometric analysis were carried out after calibration with the PeakFlow Standard Particles (ST: cod. P14825 for UV alignment and cod. P14827 for 488 nm excitation, LifeTechnology, Carlsbad, CA). For DNA denaturation and the FCM analysis by Acridine Orange (AO) metachromasia, the argon laser (Innova Coherent 90/5UV) was tuned at 488 nm with a power output of 200 mW. Green fluorescence emanating from the AO stained dsDNA was collected through a band-pass filter (BP) at 530/20 nm and a dichroic mirror at 560 nm. A long pass filter at 640 nm captured the red AO-stained ssDNA fluorescence. For the FISHIS analysis, the first argon ion laser was tuned to multiline UV (wavelength  = 353–361 nm) at 200 mW output, to excite the DAPI stained chromosomes and to generate the trigger system signal. The DAPI fluorescence emission was collected through a BP filter at 420/30 nm. The second argon ion laser (Innova Coherent 305c) was tuned at 488 nm (FITC labeling; FL3 filter BP  = 530/20 nm) or at 514 nm (Cy3 labeling; FL4  =  LP580 nm) with 400 mW power output. The FACS Vantage SE was equipped with a 70 µm flow tip, running at 27psi with a sheath fluid of 50 mM NaCl. The sample throughput was set to 400particles/sec as injected by the step motor-driven 1 ml syringe. Sorting was at a 29.7 KHz drop drive frequency, with a sorting rate of 5–20/sec in dual-sorting mode. Sorted chromosomes were collected either on glass slides for immediate identification, or in DNA Eppendorf LoBind tubes (Eppendorf, Germany) in ddH_2_O for further processing. Flow cytometric data were collected and analyzed using the software package CellQuest Pro v4.01 (BD Bioscience, San Jose, CA). As already described [9], the primary analysis gate was set on a dual parameter dot plot comprising Forward Scatter (FSC) versus FL1H (H =  signal height, DAPI fluorescence) to discriminate chromosomes from debris and chromosome aggregates. Sorting windows (**Figure 2**) were drawn on the fluorescence dot plot of FL1A (A =  signal area; DAPI fluorescence) versus FL3H (FITC fluorescence) or FL4H (Cy3 fluorescence). Sorting purity was evaluated on glass slides under a fluorescence microscope by counting one hundred FISHIS chromosomes three times per sort run, and cataloging chromosome types according to their hybridization patterns [14].

### Quantification of the FITC Fluorescence Intensity in FISHIS Labeled Chromosomes

FISHIS based on labeled (GAA)_7_-FITC was measured against a commercial set of six calibrated microbeads loaded with known quantities of fluorescein (Quantum FITC-5 MESF kit; www.bangslabs.com). The kit allows for the direct measurement and quantification of the fluorescence intensity emitted in terms of MESF. In principle, MESF allows comparison of fluorescence values among different instruments and samples, providing the same experimental conditions are guaranteed [34]. The FACS settings were: multiline UV argon ion laser with 200 mW power output, diaphragm 0, elliptical focus lens (BD 02-6092500D), FL1 photomultiplier voltage 500V, gain 1; 488 nm argon ion laser at 400 mW power output, diaphragm 4, FL3 photomultiplier voltage 600V, gain 4. A fresh suspension of Quantum FITC-5 MESF made up in LB01 chromosome isolation buffer was analyzed according to the manufacturer's instructions. Instrument settings were recorded for immediate subsequent analysis of FISHIS labeled chromosomes and nuclei. The peak median fluorescence of the Quantum beads and the sample emission values were entered into the MS Office Excel sheet available at www.bangslabs.com/products/quickcal, which is specific for each Quantum FITC-5 MESF kits preparation lot, to derive a calibration curve. Chromosome clusters were defined on a DNA fluorescence versus (GAA)_7_-FITC dot plot of pasta wheat FISHIS labeled chromosomes (**Figure 2**). MESF values were calculated for each FISHIS cluster (**Figure S4** and **Figure S5**).

### Chromosome DNA Purification and the Multiple Displacement DNA Amplification

A total of 30,000–40,000 flow-sorted chromosomes were treated with 1 µl of proteinase K solution (20 mg/ml Qiagen, Germantown, MD, USA), in 30 mM Tris-HCl pH****8 at 50°C for 18 h, loaded onto a Microcon YM-100 column (Millipore Corporation, Bedford, MA, USA) and proteinase K was removed by three washes with 450 µl H_2_O and three rounds of centrifugation at 500 g for 15 mins at 23°C. Chromosome DNA was recovered by inverting the column and centrifuging at 1,000×*g* for 3 mins, and its concentration subsequently determined by fluorimetry (Qubit1.0, LifeTechnology, Carlsbad, CA) and adjusted to 10 ng/µl. The multiple displacement DNA amplification was performed using a GenomiPhi V2 DNA Amplification kit (GE Healthcare, Chalfont St. Giles, UK) in a 20 µl reaction volume for 1.5 h at 30°C, according to the manufacturer's instructions. DNA amplification was evaluated by electrophoresis on 0.5% agarose gels with TBE (89 mM Tris base, 89 mM boric acid, 2 mM EDTA, pH****8) for 30–60 mins at 8 V/cm and 0.1 µg/ml ethidium bromide staining (**Figure S6**).

### DNA extraction

Genomic DNA was isolated from young leaves of *T. durum* cv Creso using NucleoSpin Plant II kit (Macherey-Nagel) according to the manufacturer's instructions.

### PCR amplifications using FISHIS-flow-sorted chromosome DNA

Chromosome-specific primers derived from the SSR molecular markers Xgwm136 (L5′-GACAGCACCTTGCCCTTTG-3′ and R5′-CATCGGCAACATGCTCATC-3′), Xgwm169 (L5′-ACCACTGCAGAGAACACATACG-3′ and R5′-GTGCTCTGCTCTAAGTGTGGG-3′), Xgwm285 (L5′-ATGACCCTTCTGCCAAACAC-3′ and R5′-ATCGACCGGGATCTAGCC-3′), located on chromosome 1A, 6A and 3B, respectively, were selected from the GrainGenes database (http://wheat.pw.usda.gov/cgi-bin/graingenes/browse.cgi), and were used to amplify pasta wheat DNA from different fractions of sorted chromosomes and from total genomic DNA. PCR samples were prepared in 25 µl total volume with 10 µM primers (Eurofins MWG Operon) 2× PCR master mix (Thermo Scientific K1071) using as template DNA from: 300 flow-sorted chromosomes for each 1A, 6A and 3B chromosome-specific fraction (0.2 and 0.3 ng DNA, respectively [11]); 2500 flow-sorted chromosomes each for the total A- and B-genome complement fraction; 6000 flow-sorted chromosomes for the total chromosome fraction corresponding to the whole genome, and 10 ng of genomic DNA isolated from leaves. In each experiment, a DNA 50 bp-step ladder was used (SIGMA S7025). Chromosomes were flow-sorted directly into 0.2 ml tubes filled with 3 µl H_2_O; PCR amplification conditions were: 2 mins at 94°C; 40 cycles with 30 sec at 94°C, 1 min at 60°C, and 1 min at 72°C; and a final extension step of 10 mins at 72°C. Amplification products were separated by TBE 2% agarose gel electrophoresis for 80 mins at 6 Volts/cm and 0.1 µg/ml ethidium bromide staining.

### Fluorescence microscopy

FISHIS labeled chromosomes and nuclei were observed under a Nikon Eclipse TE2000-S epifluorescence microscope equipped with a Hg100 lamp and filter sets appropriate for FITC, DAPI and Cy3 fluorescence. Individual images from each filter set were captured and digitalized using a NIKON DXM1200 color camera (Nikon Instruments Europe, B.V. Amstelveen, The Netherlands) and superimposed after contrast and background optimization using the ImageJ 1.46 free imaging software (NIH, Bethesda, MD; http://rsbweb.nih.gov/ij/index.html).

## Results

### FISHIS labeling set-up

In the first series of experiments, we defined conditions for the controlled denaturation of the DNA double helix in chromosome suspensions. Denaturation was induced by alkaline pH treatments [27], [28] of varied duration and was assessed by flow cytometric analysis of the metachromatic shift from green to red fluorescence of Acridine Orange displayed by the chromosomes. Green fluorescence indicates native dsDNA, and red fluorescence, denatured ssDNA, respectively [32] (**Figure S1a**). Chromosomes treated at pH****13 and greater showed a high ssDNA fluorescence but chromosome morphology was diminishing as treatment duration was prolonged (see HPCV estimates. **Figure S1b**). A treatment for 20 mins at pH****13 was selected as optimal for DNA denaturation and preservation of chromosome morphology.

The next set of experiments evaluated different concentrations of probe to optimize FISHIS labeling and FCM analysis, first using a (GAA)_7_ oligonucleotide labeled with FITC. The GAA microsatellite motif is highly effective in generating a FISH karyotype for wheat [20], [21], [22] providing information essentially similar to C-banding [20]. In pasta wheat, depending on the probe concentration, a number of regions (clusters of dots) ranging from two to a maximum of seven got separated from each other in a bivariate dot plot, representing the combination of the DAPI DNA fluorescence (X axis) versus the GAA chromosome-specific FITC labeling fluorescence (Y axis) (**Figure S1c**). Best results were achieved at 160 ng/ml (GAA)_7_-FITC concentration for 1 h at room temperature, which generated an intense and well defined hybridization pattern along chromosomes and nuclei in suspension (**Figure 1**) and yielded an optimal discrimination (7 regions) in the FCM analysis (**Figure S1c**, boxed area R2, and inside regions). Decreasing the probe amount, a lesser discrimination among regions was observed, while higher probe concentrations did not improve chromosome insight.

Other oligonucleotides than (GAA)_7_ were also used as single and double target probe FISHIS labeling (**Figure 1**). The (AG)_12_–Cy3 DNA probe provided a labeling pattern that permitted sorting of specific chromosomes of the wheat B genome (**Figure 1b**) and the multi-color labeling pattern was achieved by combining (GAA)_7_-FITC with either (AAC)_5_-Cy3 or (AAT)_7_-Cy3 labeling (**Figure 1**, **c** and **d**). The distribution of the (GAA)_7_-FITC sites in pasta wheat (genomes A and B) was highly reproducible (**Figure S2**) and their pattern agreed well with that observed for FISH-labeled fixed chromosomes [20], [21], [22]. Also, the fluorescence intensity produced by the (GAA)_7_ probe was proportional to the total nuclear DNA present, as shown by FCM analysis of the cell cycle DNA content changes in nuclei of pasta wheat root meristem cells (**Figure S3**). FISHIS can be completed in less than 90 mins and preparations can be stored at 5°C, extending the time for a precise FCM analysis to one month, at least.

### FISHIS at work: flow sorting of labeled chromosomes

**Figure 3:**
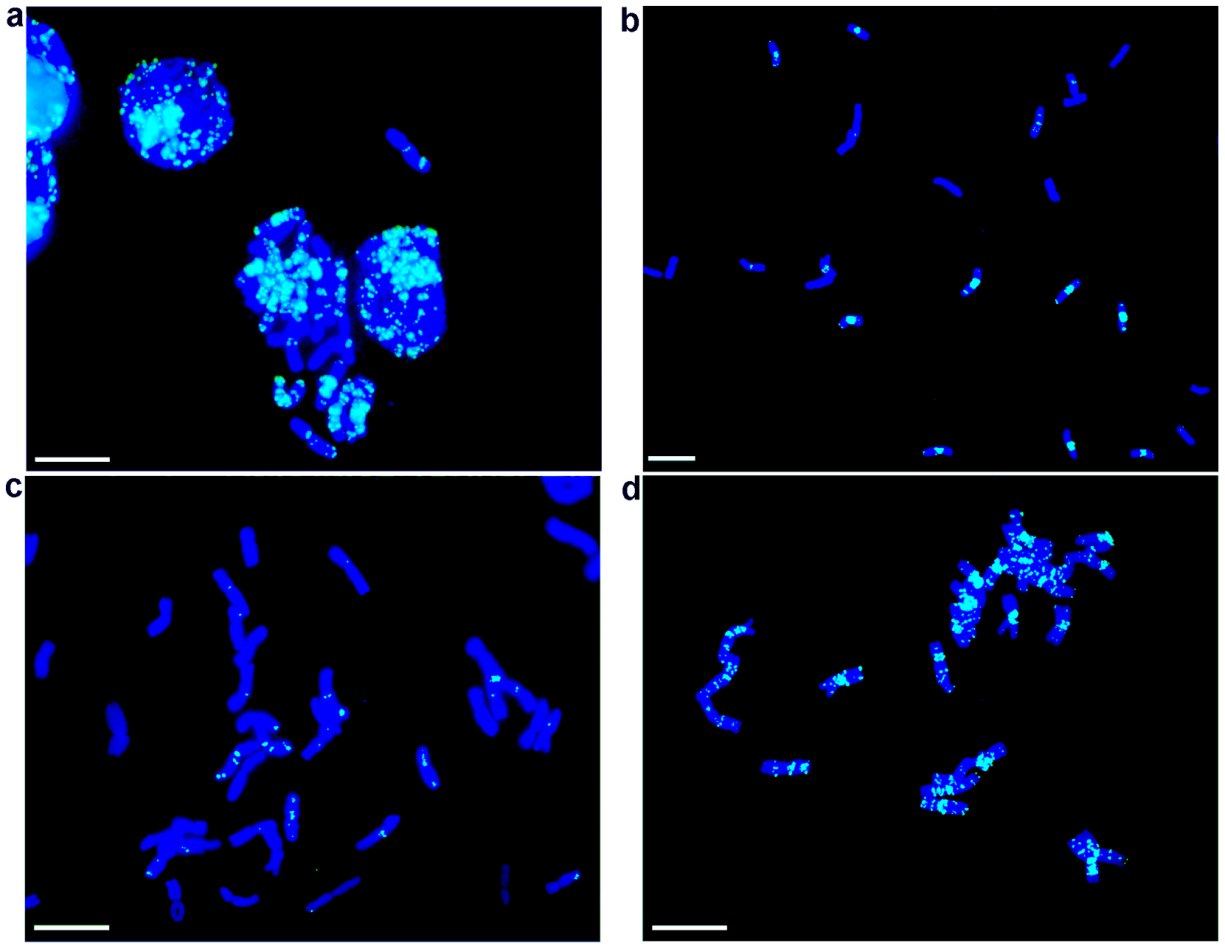
(GAA)_7_-FITC labeling and FISHIS-based flow-sorting of pasta wheat chromosomes belonging to the homeologous genomes A and B. a) Metaphase chromosomes of pasta wheat labeled by (GAA)_7_-FITC after ND-FISH on a microscope slide; b) sorting of all chromosomes after FISHIS; c and d) flow-based separation of the A- (low labeling intensity) and B-genome chromosome fractions, respectively. Bar  = 10 µm.

In pasta wheat, the standard mono-parametric flow karyotype based on DAPI staining comprises three major peaks, only one of them containing a single-type chromosome, that is 3B [22]. The FISHIS based karyotype combining DAPI and (GAA)_7_-FITC fluorescence resolved several chromosome clusters (**Figure 2**). Interestingly, differences in the abundance of the GAA motif between the two wheat genomes were clear enough (**Figure S2**) to provide easy separation of the A- and B-genome chromosomes (**Figure 3**). When chromosomes present in each cluster were assessed for overall morphology and (GAA)_7_ hybridization patterns, it became clear that the A-genome chromosomes, which are less intensely labeled than those in the B-genome, were allocated in the regions of lower (GAA)_7_-FITC fluorescence intensity in both mono- and bi-parametric flow karyograms (**Figure S4;** **Figure 2**; regions R1, R2, R3). On the other hand, the B-genome chromosomes, all of which show strong and complex (GAA)_7_ hybridization patterns, were found in regions corresponding to higher levels of fluorescence within the karyograms (**Figure S4;** **Figure 2**; regions R4, R5). At the level of individual chromosomes, FISHIS based on (GAA)_7_-FITC labeling pattern permitted flow sorting of chromosome 1A to a purity of >92%, chromosome 6A to >93% purity, chromosome 2B to >93% purity, and chromosome 3B to 99% purity (**Figure 2**).

**Figure 4:**
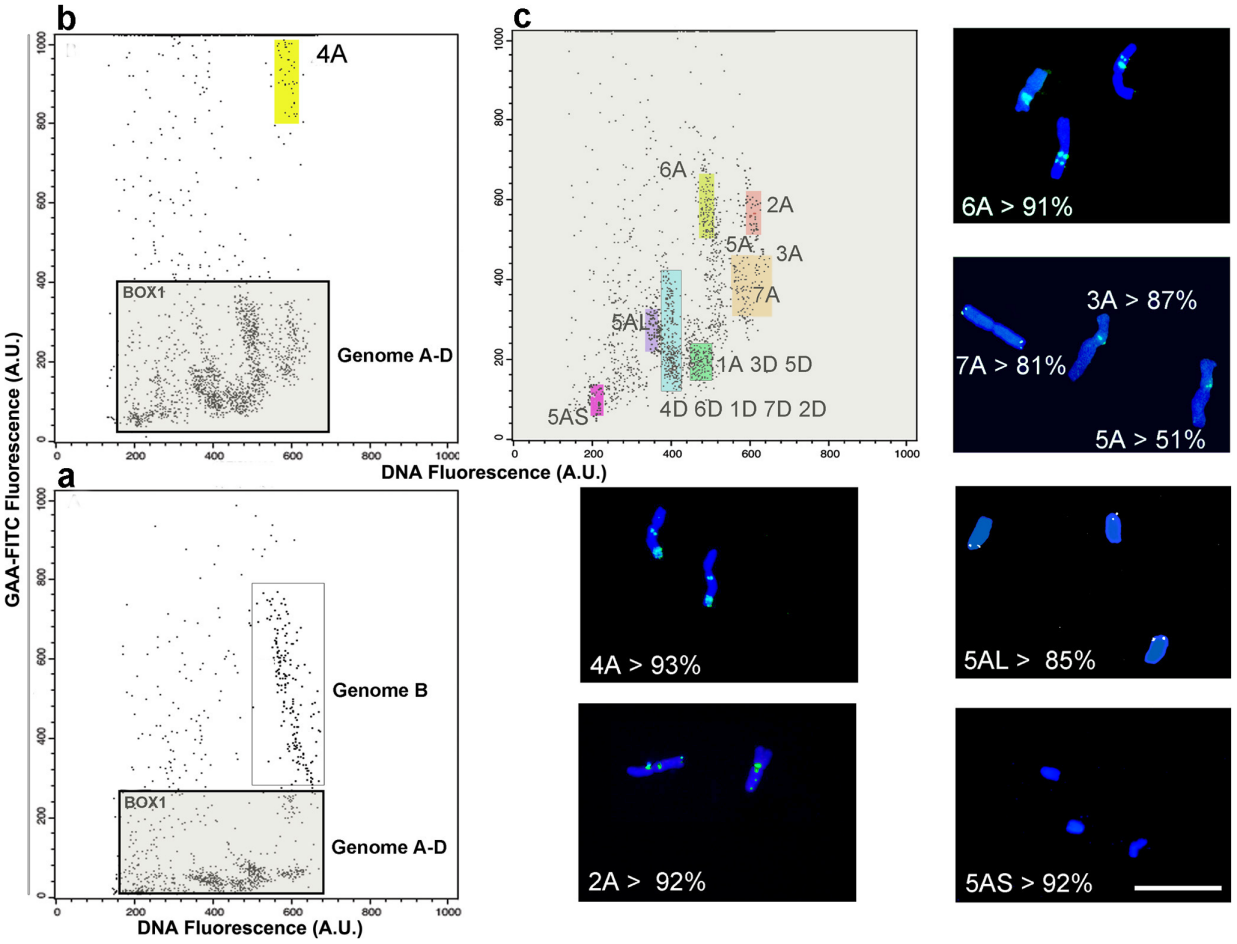
(GAA)_7_-FITC labeling and FISHIS-based flow-sorting of bread wheat chromosomes (*T. aestivum* cv Chinese Spring double ditelosomic line CSdDt5A). a) FISHIS allows the discrimination between homeologous genomes A and D (within BOX1) and B-genome; b) the increasing of the instrument sensibility towards lower fluorescence signals permits an easy flow-sorting of chromosome 4A (colored region); c) by doubling the signal amplification, all the D-genome chromosomes and the chromosome 1A can be confined into specific sorting regions (color-marked areas). Chromosomes 2A, 6A, and chromosome arms 5AS and 5AL were sortable to a high level of purity (purity percentage in Panels). Bar  = 10 µm.

In bread wheat, the standard mono-parametric flow karyotype comprehends four main peaks, but only one of these contains a single chromosome, namely 3B [10]. With FISHIS, for cv Provinciale or for line CSdDt5A (a CS cytogenetic stock previously used for the isolation and sequencing of chromosome 5A arms [35]), it was possible to discriminate between all three homeologous genomes (**Figure 4a**), and inside each genome region, specific individual chromosomes were characterized and located. **Figure 4** illustrates a detailed analysis of FISHIS in CSdDt5A, and how, by optimizing the flow cytometer set up, it becomes possible to identify specific components of the wheat genome (**Figure 4a**). As previously seen for pasta wheat, the B-genome of bread wheat can be easily recognized within the upper region of the dot plot. After optimization, it was possible to sort chromosome 4A to a purity of above 93% (**Figure 4b**, region in color). Further magnification of the relevant region in the dot plot demonstrates discrimination of all A- and D-genome chromosomes, with the exception of 1A (**Figure 4c**). Chromosomes 2A and 6A were subsequently sorted to high purity levels (>92% and >91%, respectively; **Figure 4c**). It should be noted that a further advantage of FISHIS is that chromosome purity assessment, conventionally done by an overnight FISH analysis using the sorted fraction [14], is made in real time, since chromosomes are labeled already, which means that the sorting parameters can be immediately optimized. This new feature could facilitate the standard chromosome approach, expediting whole chromosomes and single arms spotting (see telocentrics 5AS and 5AL: **Figure 4c**).

**Figure 5:**
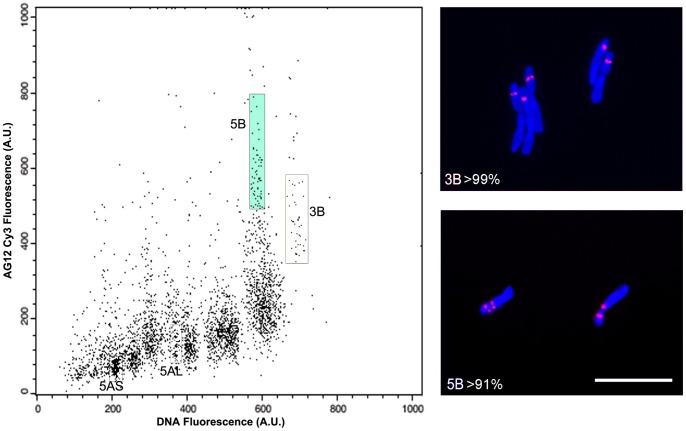
High purity flow sorting of (AG)_12_-Cy3 labeled 5B and 3B chromosomes from bread wheat line CSdDt5A. Two sorting regions can be drawn on a DAPI-DNA fluorescence versus a (AG)_12_-Cy3 FISHIS fluorescence dot plot, which enclose chromosomes 5B and 3B at a purity of 91 and 99%, respectively. Chromosome arms 5AS and 5AL are also bordered to show the high selectivity for chromosome labeling of the probe (AG)_12_, which do not alter the discrimination of the other remaining chromosomes and chromosome arms, as shown for (GAA)_7_ labeling (**Figure 4**). Bar  = 10 µm.

The FISHIS high resolution power can be corroborated by the observation that labeled chromosome 7A from bread and pasta wheat showed the same hybridization pattern as reported previously [10], [22]. In bread wheat, this chromosome exhibits distal (telomeric) bands on both arms (**Figure 4**) whereas its 7A counterpart in pasta wheat exhibits a distal band only on the long arm (**Figure S2**). For both pasta and bread wheat, the (AG)_12_–Cy3 probe revealed two strong hybridization bands on chromosome 5B, and single weak signals on chromosomes 1B, 3B, and 6B [17]. Using this probe, we were able to sort chromosome 5B and 3B to a purity level above 90% (**Figure 5**).

**Figure 6:**
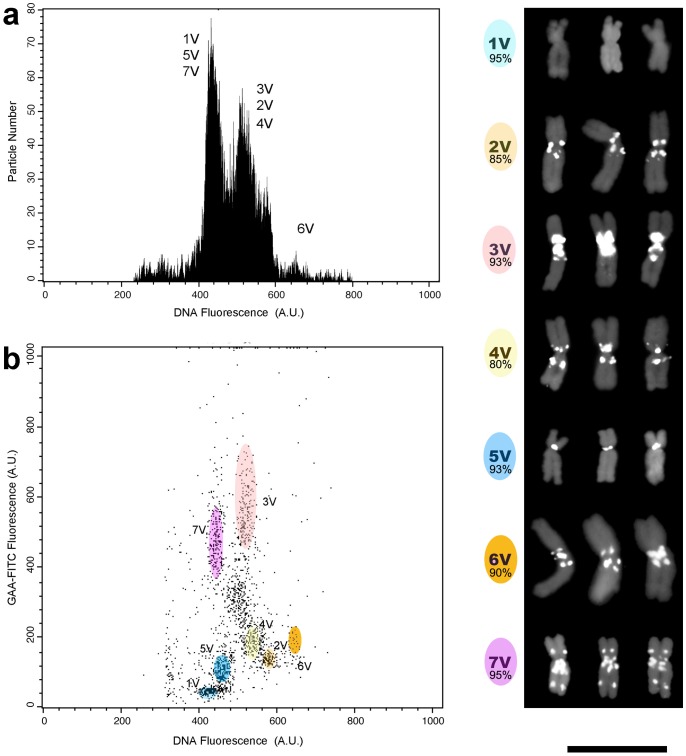
Flow karyotyping and flow sorting of each of the seven *D. villosum* chromosomes after FISHIS labeling. a) Conventional DNA content-based flow karyotyping resolves only chromosome 6V. b) FISHIS based on the (GAA)_7_-FITC labeling resolves all seven chromosomes (colored regions) which can be flow-sorted to a purity of 80–95% (purity percentage in Panels). Bar  = 10 µm.

The next series of experiments involved *D. villosum*, a wild relative of wheat used in breeding programs as a source of valuable agronomic traits [36]. Characterization of standard chromosome spreads of the *D. villosum* genome (genome V) using the (GAA)_7_ probe showed a discrimination of all seven chromosome pairs [9]. For this reason, *D. villosum* was identified as a suitable candidate for a test of the ultimate resolution power of FISHIS in the flow sorting of chromosomes, namely full discrimination of each chromosome of the genome (**Figure 6**). The standard mono-parametric flow karyotype of *D. villosum* comprises four peaks, of which only one is represented by a single chromosome, specifically 6V [9] (**Figure 6a**). As expected, FISHIS using the (GAA)_7_-FITC probe generated a dot plot karyotype in which all seven chromosomes could be individually identified and flow-sorted at the levels of purity spanning from 80% to 95% (**Figure 6b**, colored regions).

### Is FISHIS labeling specific, DNA friendly and beneficial to genomics?

Given that the ultimate aim in this work was to develop an affordable protocol by which the largest possible number of specific chromosomes of any genome could be sorted in quantities sufficient for standard applications in genomics [37], we finally examined the degree to which the method satisfied three technical performance criteria: a) the method had to be reproducible, in the sense that it consistently should result in the presence of a high correlation between the fluorescence signal strength, due to the hybridized probe, and the repetitive DNA content of a chromosome; b) the DNA of the isolated chromosomes had to be of good quality, that is, of high molecular weight; c) to demonstrate its value for downstream applications, FISHIS labeled DNA must be suitable as a template for PCR amplifications.

The issue of correlation was assessed by two independent experiments: i) measuring the ratio between DNA content (FL1) and FISHIS labeling amount (FL3) before and after DNA duplication during the cell-cycle (**Figure S3**); ii) using a process which enabled the direct measurement of fluorescence intensity (**Figure S5**) in “molecules of equivalent soluble fluorescein” (MESF) [34]. The ratio between the median fluorescence values of FL1 and FL3 measured from G_1_ and G_2_ cell-cycle phases was constant, demonstrating the direct relationship (**Figure S3**) between DNA content and FISHIS labeling. The FITC median fluorescence peak values obtained from FL3 regions drawn on a histogram (**Figure S4**: regions R1–R5) and from several sorting regions of a DNA versus GAA-FITC bi-parametric dot plot (**Figure 2**: regions R1–R5), were used to assess the (GAA)_7_-FITC fluorescence intensities associated with various chromosome clusters. Chromosomes with comparable (GAA)_7_-FITC hybridization patterns produced similar MESF values, which implies that a strong correlation exists between the MESF value and the number and intensity of hybridization sites (**Figure S5**). The value of 4,971 MESF, corresponding to chromosome 1A, was taken as the fluorescence reference level related to a chromosome that can be discriminated from background in our experimental conditions, given the flow cytometer we used for this work. Since each probe carries two FITC molecules, the MESF value of 4,971 equates to 2,485 probe sequences, or 52,195 nucleotides. Given that chromosome 1A does not show a noticeable hybridization pattern, 52 Kbp appears to represent the limit of resolution for FISHIS with the (GAA)_7_-FITC probe, at least for what concerns the non-specific labeling of pasta wheat chromosomes by this probe.

The quality of DNA, in terms of the molecular weight of the recovered chromosome DNA, was assessed electrophoretically following FISHIS (**Figure S6**). DNA from flow-sorted chromosomes was analyzed with and without (**Figure S6a**) Multiple Displacement Amplification [38] (MDA: **Figure S6b**); it was found to be of high molecular weight, because most of the DNA proved to be too large to migrate out of the low-strength agarose wells, as it is also shown for unlabeled flow-sorted chromosomes.

**Figure 7:**
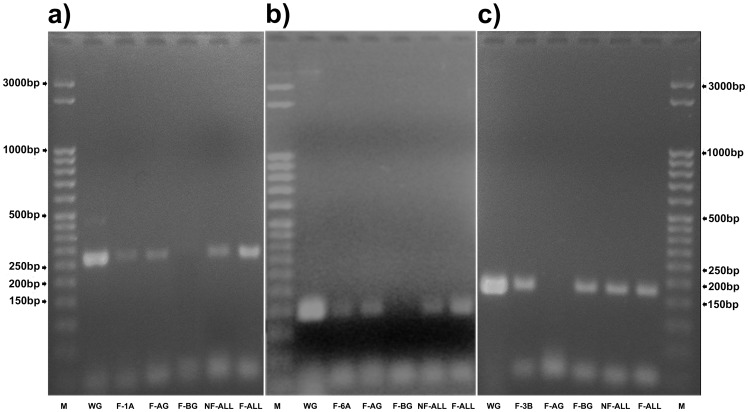
Specific PCR amplification of DNA isolated from flow-sorted FISHIS labeled pasta wheat chromosomes 1A, 6A and 3B. In all Panels: lane M: 50 bp-step ladder; lane WG: whole pasta wheat genomic DNA; lane F-AG: FISHIS labeled flow-sorted whole A-genome chromosomes; lane F-BG: FISHIS labeled flow-sorted whole B-genome chromosomes; lane NF-ALL: whole flow-sorted chromosome complement without FISHIS labeling; lane F-ALL: sorting of the whole FISHIS labeled pasta wheat chromosome complement. Panel a): chromosome 1A amplicons analysis with specific primers from the Xgwm136-1A SSR molecular marker. In lane F-1A, the DNA obtained from 300 FISHIS labeled flow-sorted 1A chromosomes has been PCR amplified showing a band which is only visible where 1A chromosome DNA is present. Panel b): chromosome 6A PCR amplification with primers from the Xgwm169-6A SSR molecular marker. In lane F-6A a specific 6A chromosome band is shown which is absent in all B-genome amplifications. Panel c): chromosome 3B PCR amplification with primers from the Xgwm285-3B SSR molecular marker. In lane F-3B, 300 FISHIS labeled 3B chromosomes have been amplified with a specific probe showing a band present in all lanes where 3B chromosome DNA is present.

Single-type FISHIS chromosome and genome fractions along with the total chromosome content of pasta wheat were flow-sorted to evaluate their suitability as a substrate for direct PCR with chromosome-specific primers. We identified and flow-sorted three pasta wheat chromosome fractions which showed very different labeling intensities (**Figure 2**), that is, 1A, 6A and 3B (**Figure S2** and **Figure S5**). The products from PCR amplifications with primers derived from the chromosome-specific SSR molecular markers Xgwm136, Xgwm169 and Xgwm285 located on chromosomes 1A, 6A and 3B, respectively, are presented and the expected amplicon bands are shown (**Figure 7**). Wheat A- and B-genome whole-chromosome complements were flow-sorted after FISHIS labeling, proving the effectiveness of the technique in providing a specific PCR probe-related pattern. No sign of contamination or DNA degradation, due to the presence of other chromosome types or to the FISHIS methodology, respectively, was detected (**Figure 7**).

## Discussion

Up until now, a routine method encompassing the high resolution power of FISH and the preparative capabilities of flow sorting has been lacking [7]. As pointed out by several authors [6], [23], [24], [25], [39], [40], [41], [42], [43], all previous attempts to directly translate FISH protocols into chromosomes in suspension have failed, due to many problems, such as chromosome clumping, paucity in suspension, poor hybridization pattern reproducibility, and loss of chromosome morphology. Here we present a straightforward wash-less method for fluorescence *in situ* hybridization of plant chromosomes in suspension (FISHIS), using synthetic, fluorescence-labeled DNA probes (**Figure 1**) that quantitatively assess specific hybridization patterns, thus allowing for precise flow sorting of individual chromosomes to high purity (**Figure 2**).

Recently, a flow cytometric analysis of human chromosomes that relied on FISH labeling in suspension by a synthetic peptide nucleic acid has been reported [44]. FISHIS allows a faster and simpler labeling of chromosomes, which in turn results in an accurate flow sorting of single-type chromosomes and whole genomes of very similar genetic constitution (e.g. homeologous genomes). FCM discrimination of FISHIS labeled chromosomes is founded on the ability to effectively hybridize DNA simple sequence repeats which are scattered all around the chromosome DNA in a non random fashion [31]. Each chromosome will then be characterized by two main features, or parameters: its total DNA content and its FISH labeling fluorescent pattern, and the combination of both parameters will be shown as single dot on a bi-parametric dot plot drawing on the flow cytometer computer screen. Looking at these clusters of dots (chromosomes), a sorting gate can be drawn to separate all the particles tagged that way, which can be sorted on a slide and immediately checked for purity and yield by microscope observation of the FISHIS fluorescence banding. As a proof of concept for whole-genome separation, we demonstrated for pasta wheat that the less intensely labeled A-genome chromosomes are clearly separated (**Figure 2**: R1-R2-R3) from the strongly labeled B-genome chromosomes (**Figure 2**: R4–R5). D-genome chromosomes from bread wheat can also be flow-sorted, with the only exception of the contamination from the 1A chromosome (**Figure 4**). Separation and isolation of constituent genomes can reduce the confounding effects of allopolyploid complexity in data analysis by Next Generation Sequencing technologies [45], [46], [47], [48], [49], [50], [51]. In the same way, high purity sorting of single wheat chromosomes 1A, 2A, 4A, 6A, 2B, 3B, 5B (**Figure 2** and **Figure 5**) and each of the seven chromosomes of *D. villosum* (**Figure 6**), represents a breakthrough and an example that extends the chromosome approach to genome sequencing beyond model crops and species. The availability of single-type chromosome-specific DNA from major species will facilitate the development of molecular markers from small amounts of flow-sorted chromosomes, and it will enable the construction of highly saturated genetic maps from specific genome regions; furthermore, it will facilitate the analysis of the haplotype in complex genomes, supporting a comprehensive gene content analysis and gene discovery [51], [52], [53], [54], [4]. The presence of a variable number of random unwanted chromosome types (e.g. 2–15%) in sorted fractions is a common feature of flow genomics [8], [35], [40], [51], [52], since high precision flow sorters have to face the interaction with the intrinsic variability of biological samples, which can indeed be envisaged, but not sorted out without increasing the discriminatory capabilities of the technique [8]. This kind of contamination in sorted fractions has been demonstrated not to invalidate NGS results [35], [40]. In this respect, FISHIS will contribute to a higher degree of discrimination, letting the isolation, at high purity levels, of all individual chromosomes of a species rely on the availability of probes that produce distinctive and specific chromosome hybridization patterns, not in terms of signal distribution but in terms of fluorescence intensity. The first successful sorting of the full chromosome complement of an eukaryote, as exemplified for *D. villosum*, can be ascribed to differences in the total fluorescence GAA bands intensity among chromosomes, even between those that show similar banding localization. Given the ubiquity across and within eukaryotic genomes of microsatellite sequences [16], [55], [56], their use as FISHIS probes offers, in principle, access to the specificity of individual nuclei and chromosomes of virtually all eukaryotes. We believe that FISHIS could contribute to the analysis and sorting of specific animal chromosomes with peculiar abnormalities, such as copy number variations and cytogenetic aberrations [57], which can be revealed by microsatellite probes. It would not only enhance analytical and preparative capabilities of flow cytometry but would also open new horizons to genomics and biotechnology.

## Supporting Information

Figure S1 **Setting the parameters for FISHIS labeling.** Flow cytometry analysis of pasta wheat cv Creso chromosome suspensions was used to optimize pH for denaturation, treatment time, and the (GAA)_7_-FITC probe concentration. a) Chromosomes were exposed to a range of pH values (8.0–13.8) for 20 mins, and the degree of DNA denaturation was assessed by the AO metachromasia (dsDNA: green, ssDNA: red). The ssDNA content increases with the increase in pH value. b) The effect of the treatment duration (0–60 mins intervals) at pH 13.0 on DNA fluorescence and chromosome yield. HPCV (Half Peak Coefficient of Variation) percentage values indicate the dispersion of chromosome DNA fluorescence intensities as the ratio of the standard deviation to the mean measured at 50% peak height. An internal standard (ST: PeakFlow cod. P14825) was included to ensure the stability and consistency of the measurements during analysis. c) A range of (GAA)_7_-FITC concentrations (0.3–640 ng/ml) were compared for FISHIS labeling efficiency. The dot plot of DAPI staining versus (GAA)_7_-FITC signal shows how the FISHIS signal intensity and specificity increased up to a probe concentration of 160 ng/ml. The boxed area R2 contains the intact FISHIS-labeled chromosomes clustered into a variable number of separated regions according labeling intensity and DNA content. Other signals derived from labeled chromatids generated during cell cycle synchronization and cell disruption are shown outside the boxed area.
(TIF)

Figure S2 **The FISHIS (GAA)_7_ labeling pattern in pasta wheat cv Creso flow-sorted chromosomes.** Flow-sorted chromosomes after (GAA)_7_-FITC labeling (green signal): A-genome chromosomes present a simpler banding pattern in respect to B-genome ones: all the chromosomes can be identified according to their labelling pattern. Four examples of each FISHIS labelled chromosome are given, confirming the consistency of the hybridization pattern. Chromosomes are counterstained with DAPI (DNA labelling, blue color). Bar  = 10 µm.
(TIF)

Figure S3 **The intensity of fluorescence emission from pasta wheat nuclei in suspension labeled by GAA-FITC (FL3) is proportional to the nuclear DNA (FL1) content.** a and b) nuclei at G_1_ and G_2_ cell cycle phases were flow-sorted after FISHIS labeling, respectively; c and d) FCM analysis of DAPI-stained (DNA fluorescence) and GAA-FITC (FISHIS) labeled pasta wheat nuclei, respectively (AU: arbitrary fluorescence units); e) the bivariate dot plot fluorescence analysis of both emissions from DAPI/GAA-FITC labeled pasta wheat nuclei demonstrate a straight correlation among the DNA fluorescence amount and FISHIS fluorescence intensity. Bar  = 10 µm.
(TIF)

Figure S4 **Fluorescence intensity histogram of GAA-FITC FISHIS labeled bread wheat chromosomes.** Chromosomes showing variable fluorescence intensities, produce a composite fluorescence distribution curve where relative median fluorescence of shown peaks (region R1–R5) underneath several chromosome types, which are discriminated at most by a DNA content (FL1) versus GAA-FITC fluorescence (FL3) bivariate dot plot (**Figure 2**).
(TIF)

Figure S5 **Comparing fluorescence intensity measurements and pattern on FISHIS labeled chromosomes.** FISHIS labelled pasta wheat chromosomes were analyzed according their FITC median fluorescence intensities and characteristic banding pattern (**Figure S4** and **Figure S2**). The small band shown on chromosome 3A (**Figure S2**) was selected as an arbitrary reference unit for band number estimation. Chromosomes with similar bands in number and/or fluorescence intensity fall into the same region (**Figure 2**: regions R1–R5). FITC median fluorescence intensities calculated from a univariate histogram (**Figure S4**) were converted to an absolute unit of fluorescence as Molecules of Equivalent Soluble Fluorochrome (MESF). MESF values should allow to assess the instrument sensitivity, to compare data among different instruments and to calculate FISHIS efficiency in terms of the amount of molecules of fluorescein bound to the sample.
(TIF)

Figure S6 **Flow-sorted FISHIS chromosomes yield High Molecular Weight DNA.** Native (a) and MDA (b) DNA from FISHIS chromosomes of pasta wheat was evaluated by agarose gel electrophoresis stained with ethidium bromide. a) lane 1: 1 Kbp ladder; lanes 2 and 3: 30 ng and 100 ng of flow-sorted FISHIS labeled chromosome DNA, respectively; lane 4: 100 ng of flow-sorted unlabeled chromosome DNA. b) lane 1: 1 Kbp ladder; lane 2: 200 ng of sorted FISHIS labeled A-genome chromosomes; lane 3: 200 ng of sorted FISHIS labeled B-genome chromosomes; lane 4: 200 ng of all unlabeled chromosomes; lane 5: HMW control DNA (GE Healthcare kit). HMW DNA did not migrate out of the wells.
(TIF)
