# Supplementary figures and images for: FISHIS: Fluorescence In Situ Hybridization in Suspension and Chromosome Flow Sorting Made Easy
Source: PLoS One. 2013 Feb 28;8(2):e57994. doi: 10.1371/journal.pone.0057994 (PMC3585268; doi:10.1371/journal.pone.0057994)

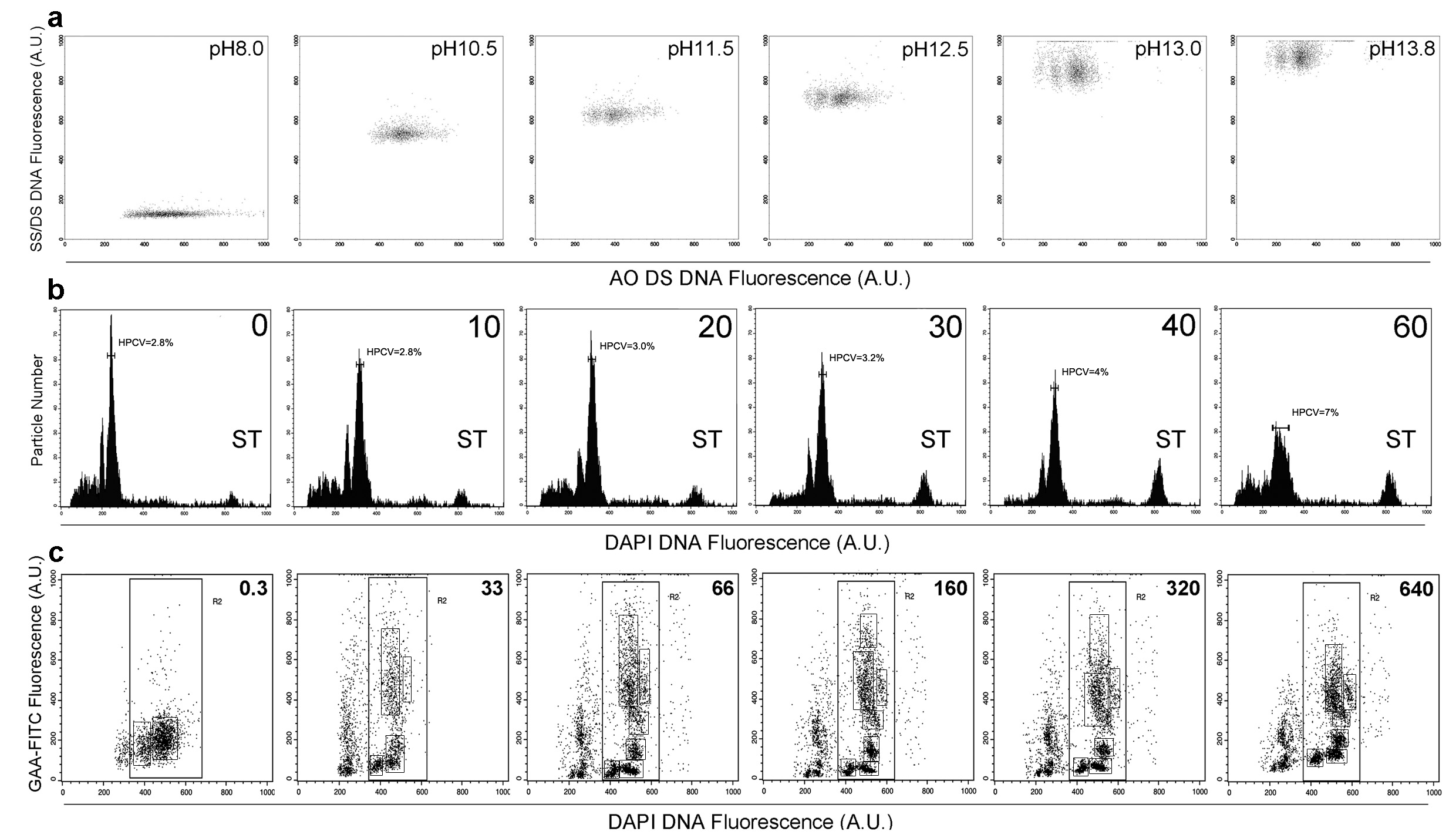

Supplement: Figure S1 — Setting the parameters for FISHIS labeling. Flow cytometry analysis of pasta wheat cv Creso chromosome suspensions was used to optimize pH for denaturation, treatment time, and the (GAA)7-FITC probe concentration. a) Chromosomes were exposed to a range of pH values (8.0–13.8) for 20 mins, and the degree of DNA denaturation was assessed by the AO metachromasia (dsDNA: green, ssDNA: red). The ssDNA content increases with the increase in pH value. b) The effect of the treatment duration (0–60 mins intervals) at pH 13.0 on DNA fluorescence and chromosome yield. HPCV (Half Peak Coefficient of Variation) percentage values indicate the dispersion of chromosome DNA fluorescence intensities as the ratio of the standard deviation to the mean measured at 50% peak height. An internal standard (ST: PeakFlow cod. P14825) was included to ensure the stability and consistency of the measurements during analysis. c) A range of (GAA)7-FITC concentrations (0.3–640 ng/ml) were compared for FISHIS labeling efficiency. The dot plot of DAPI staining versus (GAA)7-FITC signal shows how the FISHIS signal intensity and specificity increased up to a probe concentration of 160 ng/ml. The boxed area R2 contains the intact FISHIS-labeled chromosomes clustered into a variable number of separated regions according labeling intensity and DNA content. Other signals derived from labeled chromatids generated during cell cycle synchronization and cell disruption are shown outside the boxed area. (TIF) [file pone.0057994.s001.tif]

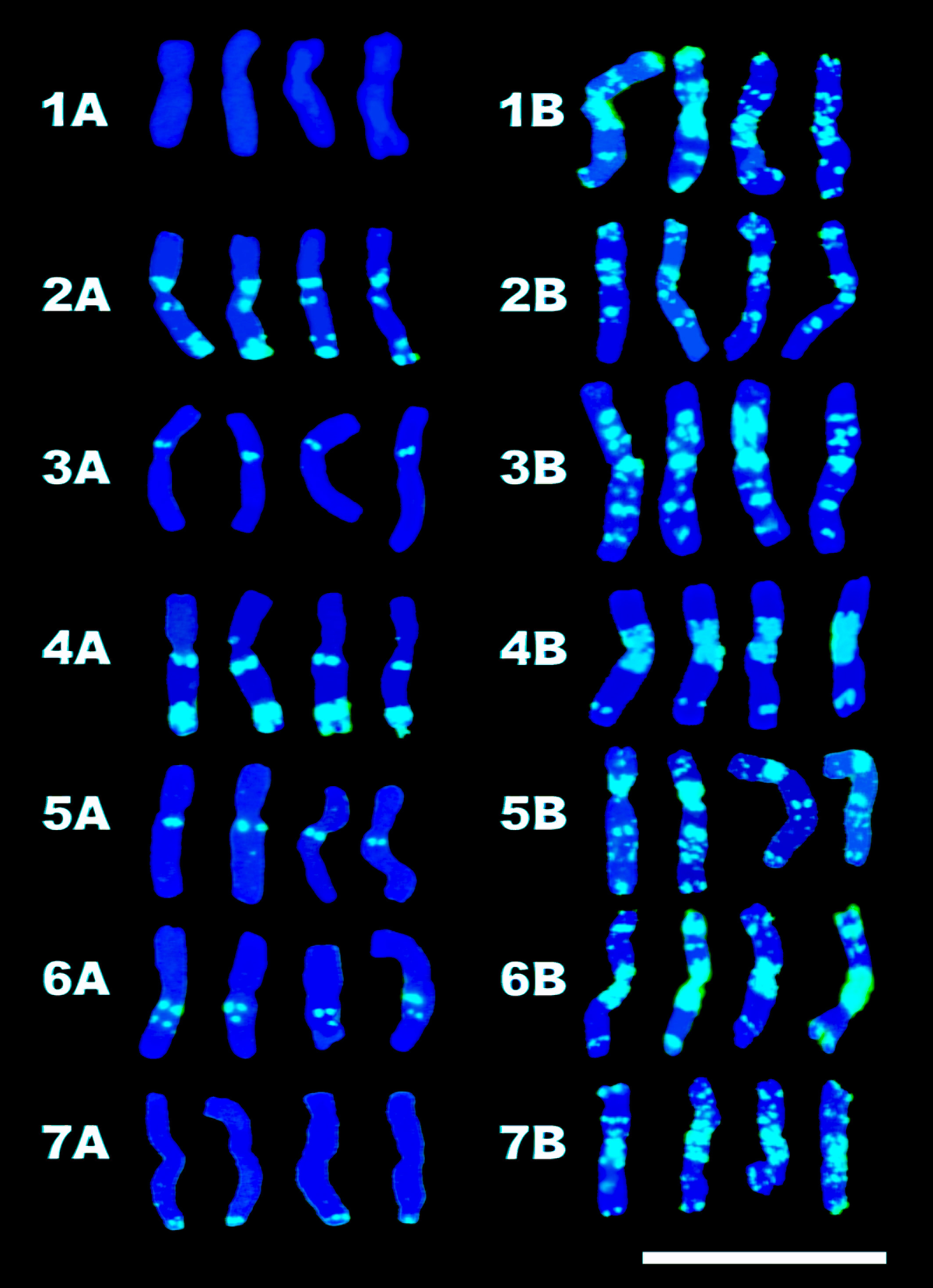

Supplement: Figure S2 — The FISHIS (GAA)7 labeling pattern in pasta wheat cv Creso flow-sorted chromosomes. Flow-sorted chromosomes after (GAA)7-FITC labeling (green signal): A-genome chromosomes present a simpler banding pattern in respect to B-genome ones: all the chromosomes can be identified according to their labelling pattern. Four examples of each FISHIS labelled chromosome are given, confirming the consistency of the hybridization pattern. Chromosomes are counterstained with DAPI (DNA labelling, blue color). Bar = 10 µm. (TIF) [file pone.0057994.s002.tif]

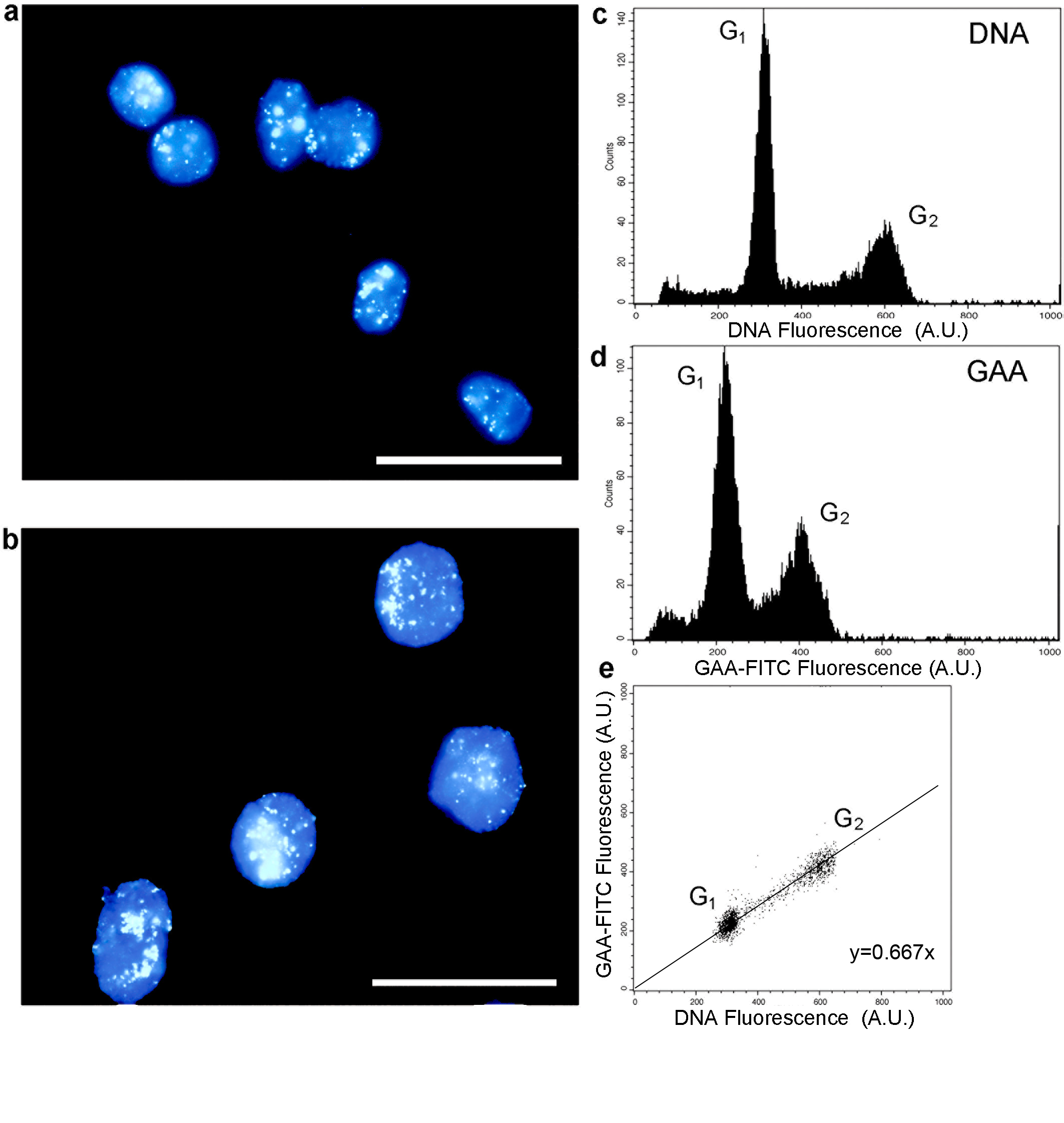

Supplement: Figure S3 — The intensity of fluorescence emission from pasta wheat nuclei in suspension labeled by GAA-FITC (FL3) is proportional to the nuclear DNA (FL1) content. a and b) nuclei at G1 and G2 cell cycle phases were flow-sorted after FISHIS labeling, respectively; c and d) FCM analysis of DAPI-stained (DNA fluorescence) and GAA-FITC (FISHIS) labeled pasta wheat nuclei, respectively (AU: arbitrary fluorescence units); e) the bivariate dot plot fluorescence analysis of both emissions from DAPI/GAA-FITC labeled pasta wheat nuclei demonstrate a straight correlation among the DNA fluorescence amount and FISHIS fluorescence intensity. Bar = 10 µm. (TIF) [file pone.0057994.s003.tif]

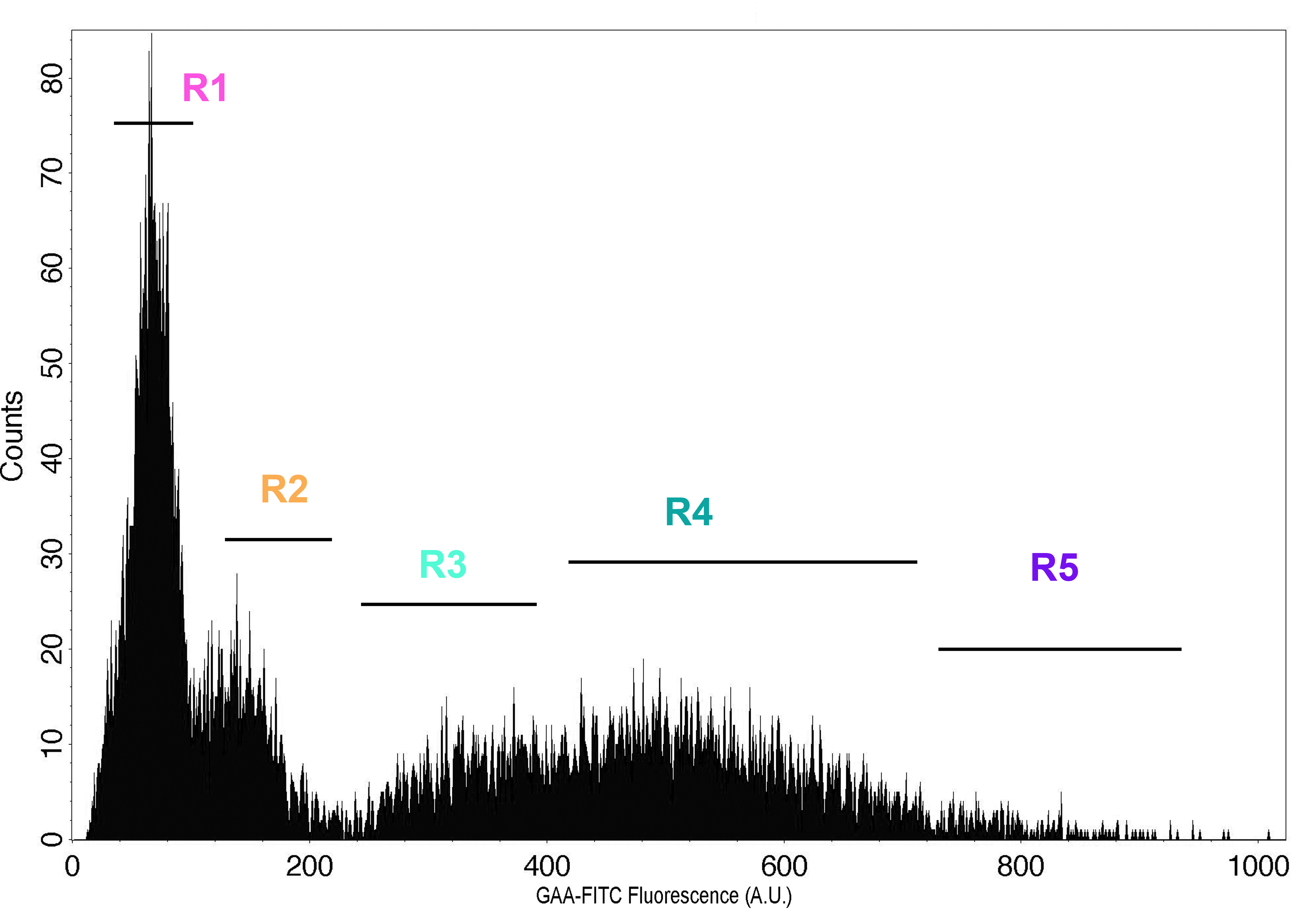

Supplement: Figure S4 — Fluorescence intensity histogram of GAA-FITC FISHIS labeled bread wheat chromosomes. Chromosomes showing variable fluorescence intensities, produce a composite fluorescence distribution curve where relative median fluorescence of shown peaks (region R1–R5) underneath several chromosome types, which are discriminated at most by a DNA content (FL1) versus GAA-FITC fluorescence (FL3) bivariate dot plot ( Figure 2 ). (TIF) [file pone.0057994.s004.tif]

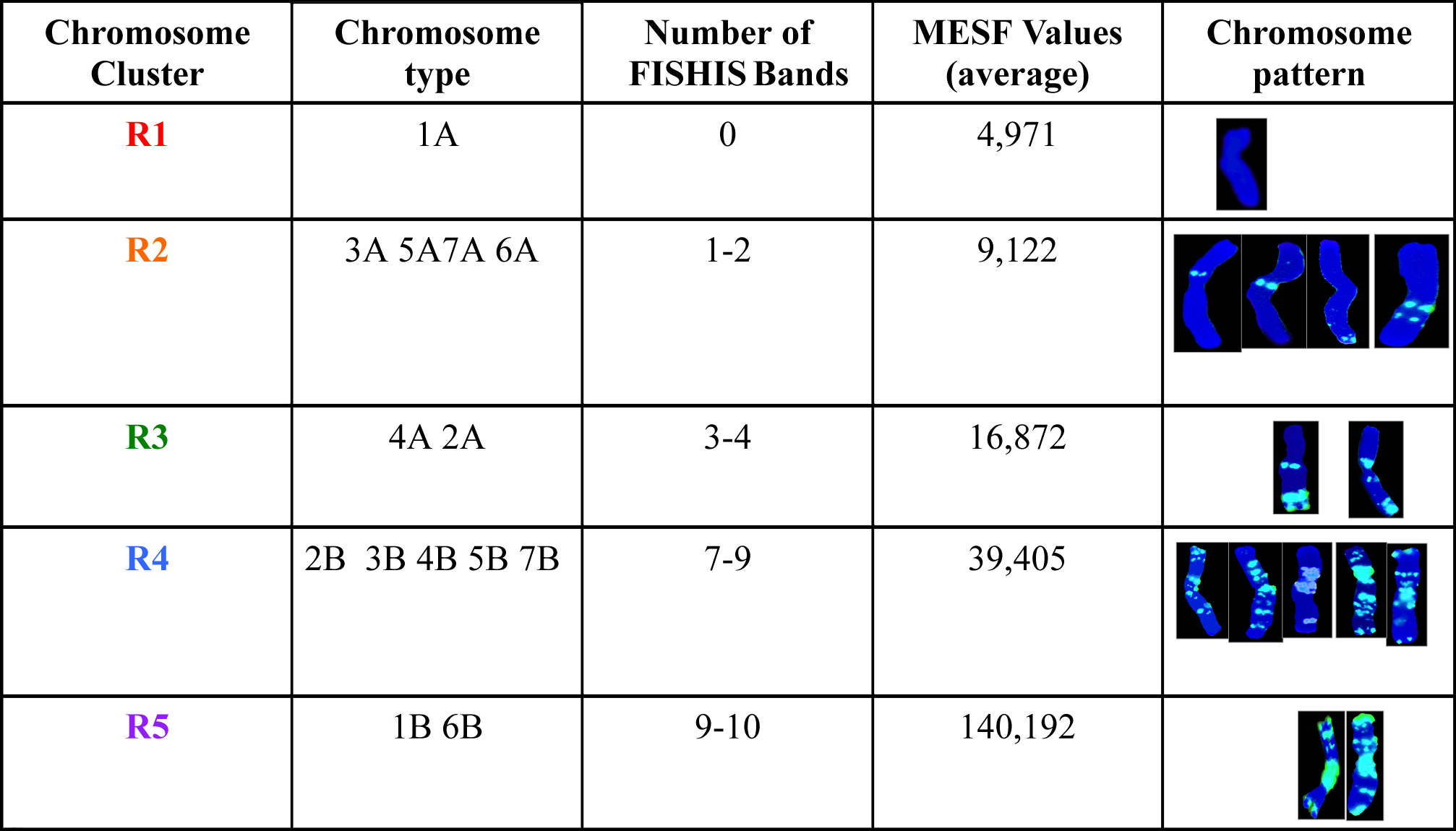

Supplement: Figure S5 — Comparing fluorescence intensity measurements and pattern on FISHIS labeled chromosomes. FISHIS labelled pasta wheat chromosomes were analyzed according their FITC median fluorescence intensities and characteristic banding pattern (Figure S4 and Figure S2). The small band shown on chromosome 3A (Figure S2) was selected as an arbitrary reference unit for band number estimation. Chromosomes with similar bands in number and/or fluorescence intensity fall into the same region ( Figure 2: regions R1–R5). FITC median fluorescence intensities calculated from a univariate histogram (Figure S4) were converted to an absolute unit of fluorescence as Molecules of Equivalent Soluble Fluorochrome (MESF). MESF values should allow to assess the instrument sensitivity, to compare data among different instruments and to calculate FISHIS efficiency in terms of the amount of molecules of fluorescein bound to the sample. (TIF) [file pone.0057994.s005.tif]
